# Editorial: Precision Medicine in Neonates

**DOI:** 10.3389/fped.2021.702760

**Published:** 2021-05-31

**Authors:** Karel Allegaert, Sinno Simons

**Affiliations:** ^1^Department of Development and Regeneration, KU Leuven, Leuven, Belgium; ^2^Department of Pharmacy and Pharmaceutical Sciences, KU Leuven, Leuven, Belgium; ^3^Department of Clinical Pharmacy, Erasmus Medical Center, Rotterdam, Netherlands; ^4^Division of Neonatology, Department of Pediatrics, Erasmus Medical Center Sophia Children's Hospital, Rotterdam, Netherlands

**Keywords:** newborn, precision medicine, individualized medicine, neonatal intensive care, empirical medicine

## Introduction

**Figure 1:**
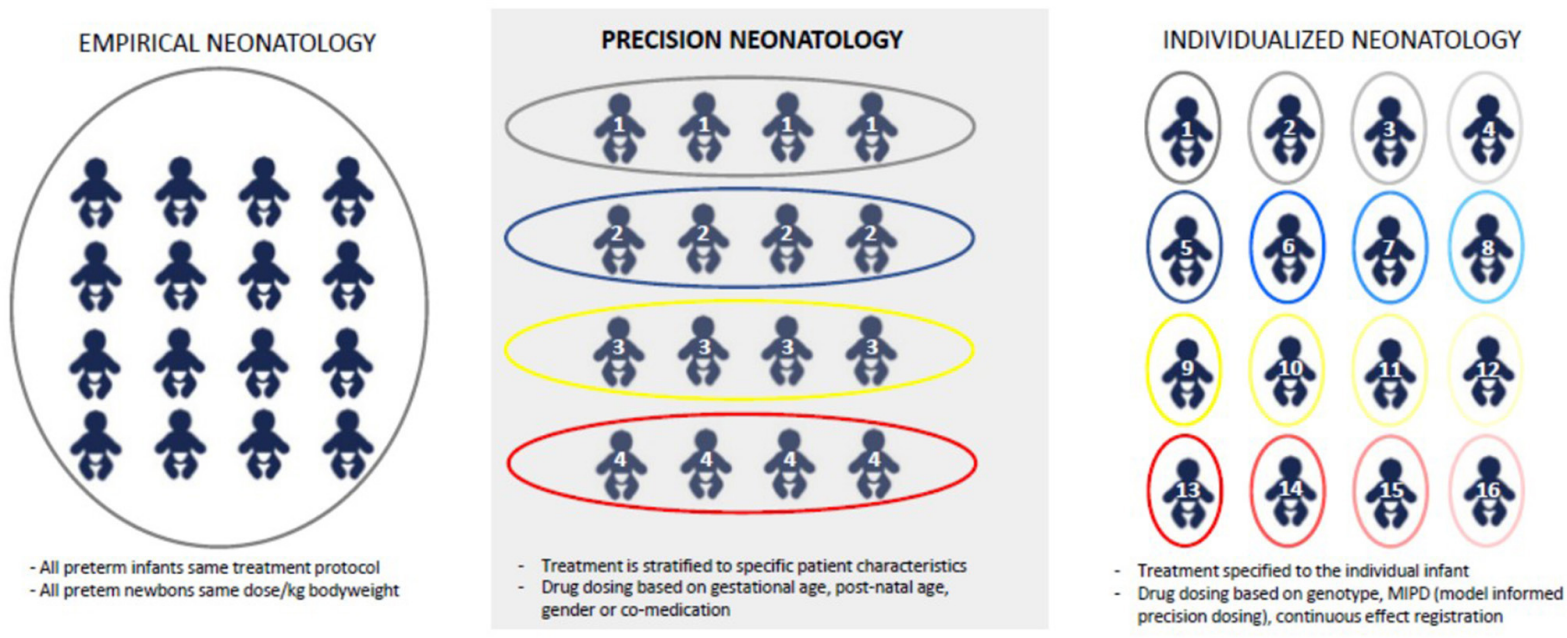
Different concepts related to empirical medicine, precision medicine, or individualized medicine.

Precision medicine can be defined as a structured approach to treat or prevent specific diseases based on inter-individual variability in genes (like polymorphisms), diseases (like gender, co-morbidity), environment (like drug exposure, nutrition), or lifestyle (like stress) (1). This concept also holds the promise to improve management (prevention or treatment) and subsequent outcome in critically ill newborns. Precision medicine (*subgroup approaches*) hereby serves as go between empirical (*one treatment fits all*) or stratified medicine (e.g., *disease state or sex*), and individualized (*every newborn is unique*) medicine (Figure 1).

With this Frontiers special issue, we intended to increase awareness among caregivers that precision medicine has a proven track record in e.g., oncology, and is likewise, also very promising to improve outcome in (pre)term neonates. Unfortunately, neonatal medicine is lagging behind in implementing this concept. To further quantify this, a PubMed for “precision medicine” on April, 23 2021 resulted in 64,080 hits, with an exponential increase from 2010 (>1,000 hits/year) onwards, while “precision medicine + newborn” only resulted in 826 hits, with a delayed increase from 2015 onwards.

This reflects the relevance of this focused Research Topic on precision medicine in neonates, as the concepts related to precision medicine and the anticipated gains in clinical outcome matter to (pre)term neonates: the neonatal community (care providers, clinical researchers, and parents) should move from one “uniform” protocol for every newborn toward precision driven, patient tailored treatments and support (2). Its feasibility and relevance are illustrated in the papers included, clustered in papers on respiratory diseases, neurology, colonization and infectious diseases, or macro- and microcirculation related (hemodynamics, renal, and retinal) diseases.

## Respiratory Diseases

Bronchopulmonary dysplasia (BPD) remains the most common complication of preterm birth with long lasting sequelae. Most intervention studies to prevent BPD were conducted on a population level, without “precision concepts” like variation in clinical and biological diversity, genetic predisposition or environmental factors. In a review paper, Onland et al. explored potential approaches to implement precision concepts in neonatal BPD care, summarizing the available evidence on electrical impedance tomography and electromyography of the diaphragm, genomic variation in caffeine metabolism, and volatile organic compound analysis in exhaled breath (Onland et al.).

Two other original studies focused on outcome and risk stratification for two medical interventions for respiratory distress syndrome (RDS), i.e., the need for a second dose of surfactant or nitric oxide. Preterms in need of a second dose of surfactant for RDS had identifiable risk factors (growth restricted, outborn, no antenatal steroids). Interestingly, this more severe RDS (reflecting the need for a second dose), phenotype was associated with lower survival, but with similar outcome in survivors (Greiner et al.). While a second dose of surfactant (up to 300 mg/kg) is still standard of care, nitric oxide remains much more controversial based on the available evidence on benefit/(long term) outcome data (Vieira et al.). The current analysis hereby paved the way for a precision medicine approach within this population by providing evidence that a specific subgroup of preterm neonates (pulmonary hypertension present, prolonged rupture of membranes, and antenatal steroid exposure) had better outcome.

## Neurology

Despite a machinery of tools (ultrasound, Magnetic Resonance Imaging, electro-encephalography, Near Infrared spectroscopy, general movements) to assess neurological (dys)function during neonatal stay, predictive performance for the subsequent individual neurodevelopmental, long-term outcome remains poor. Machine learning approaches were suggested as powerful approach to change performance of prediction and prognosis in the field of neonatal neurology (Tataranno et al.).

## Colonization and Infectious Diseases

The evidence of lung microbiota dysbiosis and the applications of probiotics to prevent BPD were examined and discussed (Yang and Dong). Based on the same gut-lung axis concept and BPD risk, the correlation between intestinal and pharyngeal microbiota was explored in 13 premature neonates. While intestines and pharynx shared some microbiota (like streptococcus), both sites also had unique profiles, so that linking of data on microbiota to gut-lung crosstalk outcome data (like BPD) should also consider the site of collection (Yang et al.).

Colonization patterns are obviously also affected by our practices and antibiotic prescription (when, what and how) (3). Keij et al. therefore called for a stratified management of bacterial infections in late preterm and term neonates. Differences in disease susceptibility, disease severity, immune response and pharmacokinetics and -dynamics should be considered to develop treatment algorithms for (suspected) sepsis, to assist clinicians in their decisions on to initiative or continue antimicrobial therapy in specific subpopulations (Keij et al.).

## Macro- and Microcirculatory Diseases

Individualized hemodynamic management, tailored to the cardiovascular (patho)-physiology, and clinical characteristics of each individual patient or subgroups is another promising application of precision medicine. De Boode applied this concept to three clinical syndromes commonly observed in the neonatal unit: patent ductus arteriosus, shock, and hypotension and persistent pulmonary hypertension of the newborn (De Boode).

Renal precision medicine also holds the promise to support decision making on pharmacotherapy, signal detection of adverse (drug) events and to improve accuracy prediction of short- and long-term prognosis. Recent advances in this field (acute kidney definition development, specific findings in extreme low birth weight infants, and in neonates undergoing whole body hypothermia following asphyxia) and the potential clinical impact (secondary prevention and feasibility of nephrotoxicity risk mitigation) were discussed (Allegaert et al.). Along the same line, but integrating preterm birth with asphyxia, renal function profiles in preterm neonates with birth asphyxia within the first 24 h of life were described. Different profiles between preterms with or without asphyxia were identified. This facilitates identification of relative risks to develop renal impairment, and brings this population closer to precision medicine, and subsequent needed studies on long-term cardiovascular and renal outcome (Zhang and Zeng).

Finally, the retinal circulation and the related retinopathy of prematurity (ROP) forms another relevant morbidity in former preterms. The extent of temporal avascular area of the retina (disc diameter) and duration of mechanical ventilation at first examination of the newborn (4–6 weeks postnatal life) predicted the subsequent need for an ROP-related intervention during neonatal stay. Such indicators can be used in a risk calculator to predict the need for treatment or to power preventive studies (Chaves-Samaniego et al.).

Overall the different papers added ideas and some provided small pieces of the puzzle that we need for precision medicine. In most areas stratified medicine is still work in progress and it still quite far away from where we aim to be in neonatology. We are confident that the illustrations provided in this Research Topic will further boost the clinical research on precision medicine concepts as a powerful tool to further improve neonatal management, quality of care, and outcome.

## Author Contributions

All authors contributed to the Editorial and agreed on the final version.

## Conflict of Interest

The authors declare that the research was conducted in the absence of any commercial or financial relationships that could be construed as a potential conflict of interest.
